# Pandemic-induced healthcare shifts: an observational analysis of maternal and neonatal outcomes in adolescent pregnancies

**DOI:** 10.3389/fmed.2024.1458719

**Published:** 2024-10-15

**Authors:** Orly Grobeisen-Duque, Oscar Villavicencio-Carrisoza, Carlos Daniel Mora-Vargas, Carolina Penelope Arteaga-Lopez, Maria Guadalupe Martinez-Salazar, Alejandro Rosas-Balan, Moises Leon-Juarez, Hector Flores-Herrera, Veronica Zaga-Clavellina, Ma Guadalupe Aguilera-Arreola, Addy Cecilia Helguera-Repetto

**Affiliations:** ^1^Departamento de Inmunobioquímica, Instituto Nacional de Perinatología Isidro Espinosa de los Reyes, Ciudad de Mexico, Mexico; ^2^Escuela Nacional de Ciencias Biologicas del Instituto Politecnico Nacional, Ciudad de Mexico, Mexico; ^3^Instituto de Ciencias de la Salud, Universidad Autónoma del Estado de Hidalgo, Pachuca, Hidalgo, Mexico; ^4^Coordinación de Medicina de la Adolescente, Instituto Nacional de Perinatología Isidro Espinosa de los Reyes, Ciudad de Mexico, Mexico

**Keywords:** pandemic, pregnancy, adolescence, urinary tract infection, gestational weight gain, cervicovaginitis, neonatal outcome, maternal outcome

## Abstract

**Introduction:**

The COVID-19 pandemic caused widespread changes in healthcare delivery, particularly affecting vulnerable populations such as pregnant adolescents. These patients faced additional challenges, including developmental and gestational changes, stress from isolation, and altered healthcare access, which may have impacted the incidence and prevalence of maternal and neonatal complications. This study aims to compare maternal and neonatal outcomes in adolescent pregnancies before and during the pandemic, focusing on how shifts in healthcare delivery influenced these outcomes.

**Methodology:**

A retrospective cohort study was conducted, including 340 adolescent pregnant patients who received prenatal care at a tertiary care institution. Patients were divided into two groups: pre-pandemic (*n* = 209) and pandemic (*n* = 131). Maternal data, including pre-BMI and gestational weight gain (GWG), were collected to evaluate maternal and neonatal outcomes. Statistical analysis was performed using chi-square tests, Fisher’s exact tests, and odds ratio (OR) calculations.

**Results:**

The pandemic group showed a statistically significant increase in cesarean deliveries (*p* = 0.002; OR = 1.99) and cervicovaginitis, particularly caused by Ureaplasma spp. Conversely, the pre-pandemic group had higher rates of psychoactive substance use, maternal urinary tract infections, and neonatal transient tachypnea. In the pandemic group, overweight pre-gestational BMI and cervicovaginitis were more prevalent in patients with adequate GWG, while inadequate GWG was associated with an increased risk of urinary tract infection (UTI). A significant association between pre-gestational overweight/obesity and excessive GWG was also observed (*p* < 0.05).

**Conclusion:**

The COVID-19 pandemic altered both healthcare delivery and maternal and neonatal outcomes in adolescent pregnancies. Changes in healthcare access, isolation, and shifts in medical management during the pandemic resulted in higher cesarean rates and infection rates among pregnant adolescents. These findings underscore the need for adaptable, resilient healthcare systems capable of maintaining comprehensive care even in the face of global crises. Further studies are needed to explore long-term effects on adolescent maternal and neonatal health.

## 1 Introduction

The emergence of the COVID-19 pandemic in 2020 posed a significant threat to global health, impacting personal well-being and the healthcare system as a whole (1). Numerous studies have elucidated the relationship between SARS-CoV-2 infection and its variants, and various health outcomes and susceptibilities worldwide. However, there is a paucity of literature exploring the outcomes and distinct patterns observed when comparing the periods before, during, and after the pandemic. Beyond the direct effects of COVID-19 infection, the pandemic has catalyzed significant shifts in healthcare utilization and patient behavior. Factors such as increased healthcare burden, reduced healthcare-seeking behavior due to fear or social isolation, and disruptions in routine care have collectively influenced health outcomes, particularly evident in cases of pregnancy (2).

During pregnancy, the body undergoes several changes and adaptation processes to enable normal fetal development. These anatomical, immunological, and hormonal changes aim to support the normal development of the fetus and protect both the mother and the fetus from adverse outcomes (3, 4). However, every pregnancy carries inherent risks and may not be exempt from complications (5–7). In the case of pregnant adolescent patients, there is an increased risk for adverse outcomes, necessitating specialized medical care. This increased risk stems from the simultaneous occurrence of two distinct adaptation and developmental processes: one related to pregnancy, and the other to the normal changes expected in puberty and adolescence (8). In Mexico, 18.5% of all births are to adolescent mothers, who, as pediatric patients, require specialized gynecological and obstetric care. Pediatric gynecology is essential for ensuring the health and fertility of girls, adolescents, and young women, particularly in cases with adverse outcomes (9, 10).

The COVID-19 pandemic changed the paradigm of how the healthcare system is supposed to respond to urgent and non-urgent cases. This shift involved implementing physical measures such as the use of personal protection equipment, maintaining physical distance, minimizing physical contact unless necessary, and isolation. Additionally, the burden of the COVID-19 cases and the reduced number of consultations to mitigate transmission affected the healthcare system’s ability to address non-urgent health complaints (11, 12).

Antenatal care is one of the most important pillars for ensuring a healthy pregnancy through prevention and prompt respond to detected conditions. However, during the pandemic, the effectiveness of antenatal care was compromised by fewer and shorter consultation, as well as the shift to the non-face-to-face consultations aided by telemedicine technology (11, 12). The implementation of telemedicine, brings significant benefits such as increased convenience, reduced travel time, less exposure to infectious diseases, and the ability to monitor patients continuously through certain platforms. Telemedicine also offers the potential to customize and individualize care through advanced software, tailoring treatments, and follow-ups to each patient’s specific needs. However, this shift also underscored the need for improved protocols, regulations, and software to enhance patient follow-up and monitoring. In low-income countries, limited access to smartphones and digital technology may create barriers to healthcare access. While younger generations may adapt more easily to digital healthcare, the transition to a digital era for middle-aged populations could lead to lower usage and adherence to follow-ups and treatments (13).

Patters seen before, during and after the pandemic also indicated a different incidence of diseases, from infections to mental health diseases, which have impacted in the overall patient health. Isolation played a significant role in shaping these patterns, emphasizing the importance of studying diseases during this time to understand their natural history and pathophysiology (14).

The onset of the COVID-19 pandemic in 2020 represented a profound global health crisis, disrupting personal well-being and fundamentally altering healthcare delivery systems worldwide. In addition to the direct pathogenic effects of SARS-CoV-2, the pandemic induced significant changes in healthcare-seeking behavior and clinical management protocols, particularly affecting vulnerable populations such as pregnant patients (15).

To achieve a comprehensive understanding of these impacts, we conducted a comparative analysis of the effects of the COVID-19 pandemic on health outcomes in adolescent pregnant patients. This included analyzing a range of risk factors, such as psychoactive substance use and pregestational BMI, as well as maternal outcomes like chorioamnionitis, and gestational weight gain (GWG), and neonatal outcomes including conditions related to birth weight and prematurity.

To compare the impact of the COVID-19 pandemic, we used December 2019 as the cutoff, marking the diagnosis of the first cases in Wuhan, Hubei, China. We divided our study groups into two main categories: the “pre-pandemic group,” and the “pandemic group.” By examining some variables across the distinct periods, we aim to elucidate how the pandemic has shaped maternal and neonatal health outcomes in adolescent pregnancies.

## 2 Methodology

### 2.1 Study population and group definitions

This study was conducted in accordance with the Declaration of Helsinki and approved by the Institutional Review Board and Ethics Committee of Instituto Nacional de Perinatología Isidro Espinosa de los Reyes (INPer), protocol code 2017-3-131, approved on February 7, 2018. Informed consent for the use of clinical data was obtained from all subjects involved in the study.

This is a retrospective, observational, cross-sectional study focused on adolescent pregnant women who attended the INPer, Mexico, for prenatal care and delivery of their babies from January 2018 to December 2023. Inclusion criteria: Pregnant adolescents aged 12–17 years, gestational age of 19.6 weeks or less, consistent prenatal care (multidisciplinary approach) leading to delivery at the Institute and complete electronic medical records, including pregestational maternal weight. The exclusion criteria were: patients over 17 years old, gestational age exceeding 19.6 weeks, incomplete electronic records, patients who did not receive prenatal care and deliver at the Institute and pregnancies beyond the 20th week due to insufficient information for comprehensive pregnancy development and follow-up, and women that discontinued their complete and specialized care. A gestational age of 19.6 weeks was pivotal in ensuring that patients received consistent medical care throughout their pregnancy. The most adverse outcomes at our Institute are typically observed in patients referred from other medical centers for delivery or during the late third trimester. Although we receive approximately 250–300 pregnant adolescents each year, not all meet the inclusion criteria. Therefore, we examined the entire population attending the adolescence clinic for the selection. For our sample size calculation, we used Epi Info™ version 7.2.6.0, selecting the “Unmatched Cohort and Cross-Sectional Studies” method. We set the parameters to a 95% two-sided confidence level, 95% power, and an expected outcome rate of 20% in the post-pandemic group. Using the Kelsey approach, the estimated sample sizes were 78 for the post-pandemic group (cases) and 156 for the pre-pandemic group (controls). However, we opted to recruit a larger sample size, ultimately including 209 pre-pandemic patients and 131 post-pandemic patients. To validate our final sample size, we performed a *post hoc* power analysis using our total sample size with G*Power 3.1.9.4 for χ^2^ tests (as we did for OR calculation). We found that, assuming a pandemic effect of 20–30%, our power ranged from 0.82 to 0.99.

The study included patients receiving comprehensive and specialized care from a multi-disciplinary team, including nutritionists, psychologists, medical doctors, obstetricians, and maternal-fetal specialists. Accordingly, all analyzed variables were obtained from electronic records. For sensitive psychological and psychiatric data, questionnaires and interviews were conducted, with specialists providing follow-up care for patients who required it.

Group definitions: Patients were divided into two groups:

1. Pre-pandemic group: Patients which babies were delivered between January 2018 and December 2019.
2. Pandemic group or post-pandemic-group: Patients which babies were delivered between January 2020 and December 2023, encompassing the pandemic and post-pandemic periods. This division aimed to explore the effects of the COVID-19 pandemic on maternal and neonatal health. We did not analyze COVID-19 infection because diagnostic testing was not conducted until May 2020, and after 2021, only symptomatic patients were tested. Additionally, vaccination status was not considered, as vaccination for pregnant women in Mexico began in May 2021, while our study includes patients from January 2020.

Additionally, we analyzed the impact of GWG on maternal and neonatal health within the pandemic group, based on previous findings in the pre-pandemic group, which indicated associations between inadequate GWG, intrauterine growth restriction (IUGR), and low birth weight (8). For this analysis, the pandemic group was subdivided into three categories: inadequate GWG, adequate GWG, and excessive GWG, following the guidelines of the Institute of Medicine and the recommendations of the American College of Obstetricians and Gynecologists (16).

### 2.2 Maternal and neonatal outcomes analysis

To assess the impact of the COVID-19 pandemic on the health of our adolescent patients, we compared the two defined groups and analyzed a range of variables, including psychoactive substance use, pregestational BMI (categorized as underweight <18.5, normal 18.5–24.9, overweight 25–29.9, and obesity >30) (17), preeclampsia, diabetes mellitus type 1 (DM1), cesarean delivery rates, UTI, and CVV. Some other maternal outcomes such as premature rupture of membranes, chorioamnionitis, and GWG were also assessed, based on the guidelines of the Institute of Medicine and recommendations of the American College of Obstetricians and Gynecologists (16).

Neonatal outcomes examined included sepsis, meningitis, respiratory distress syndrome, transient tachypnea, intrauterine growth restriction, and birth weight (categorized according to the World Health Organization: low weight < 2,499 g, adequate 2,500–3,999 g, macrosomia >4,000 g) (18, 19) Gestational age was categorized as preterm (less than 37 completed weeks), term (37–41.9 weeks), and post-term (>42 weeks) (19). Neonatal sex was recorded for descriptive purposes only.

### 2.3 Clinical data search and statistical analysis

Sample size calculations were performed using EpiInfo version 7.2.6.0, employing kelsey’s and Fleiss’s method with continuity correction (CC) to refine our estimates. *Post-hoc* validation estimates were performed using G*Power version 3.1.9.4.

Statistical analyses were performed using IBM SPSS Statistics 27 (SPSS Inc., Chicago, IL, United States). Percentages and measures of central tendency were calculated. One-way ANOVA was used for parametric variables with a *p*-value <0.05 indicating statistically significant differences between groups (suggesting a non-homogeneous population). Comparisons between non-parametric variables were made using the chi-square test, with a significance level of *p* < 0.05. For comparisons involving five or fewer data points, the Fisher’s exact test was used, with a significance level of *p* < 0.05. A *p*-value <0.05 indicates a significant association with our independent variables. We had only one missing data point for neonatal sepsis, which was not included in the statistical analysis. The limited missing data in the study results from excluding patients with incomplete medical records. Risk was assessed using odds ratio (OR) analysis based on 2 × 2 contingency tables, along with a 95% confidence interval (95% CI). Graphs were generated using the ForestPloter 1.1.1 package on the R platform.

## 3 Results

As of December 2023, 340 adolescent pregnant patients who received prenatal care and delivered their babies at the INPer were selected for the study, divided into pre-pandemic patients from January 2018 to December 2019 (*n* = 209) and pandemic from January 2020 to December 2023 (*n* = 131) patients. The average age of the studied population was 15.8 years old for pre-pandemic patients and 15.7 years old for pandemic patients, with no statistically significant difference (*p* = 0.461), this demonstrates that both groups are homogeneous, making our comparisons valid. All the demographic and materno-neonatal outcomes are described in Table 1.

**Table 1:** Overview of demographic and clinical profiles in mothers and neonates by pre-pandemic and pandemic groups.

			Pre-pandemic *n* = 209	Pandemic *n* = 131
			Mean ± SD or No (%)	Mean ± SD or No (%)
Maternal	Age		15.8 ± SD 0.98	15.7 ± SD 1.06
	Pregestational BMI	Underweight	27 (12.92%)	17 (12.98%)
		Normal	144 (68.90%)	92 (70.23%)
		Overweight	28 (13.40%)	17 (12.98%)
		Obesity	10 (4.78)	5 (3.82)
	GWG	Inadequate	74 (35.4%)	54 (41.2%)
		Adequate	86 (41.1%)	52 (39.7)
		Excessive	49 (23.4%)	25 (19.1%)
	Preeclampsia		21 (10.05%)	8 (6.11%)
	DM1		1 (0.48%)	1 (0.76%)
	Cesarean section		75 (35.89%)	69 (52.67%)
	Eutocic birth		134 (64.11%)	62 (47.33%)
	Premature rupture of membranes		36 (17.22%)	32 (24.43%)
	Psychoactive substance use		18 (8.61%)	3 (2.29%)
	Chorioamnionitis		5 (2.39%)	0 (0.00%)
	Urinary tract infection		78 (37.32%)	28 (21.37%)
	Cervicovaginitis		137 (65.55%)	105 (80.15%)
	*Ureaplasma* spp.		104 (49.76%)	85 (64.89%)
	*Candida* spp.		26 (12.44%)	25 (19.08%)
	*Streptococcus agalactiae*		2 (0.96%)	2 (1.53%)
	*Gardnerella vaginalis*		41 (19.62%)	19 (14.50%)
	*Mycoplasma* spp.		13 (6.22%)	12 (9.16%)
	IUGR		24 (11.48%)	23 (17.56%)
Neonatal	Sex	Female	99 (47.36%)	63 (48.09%)
		Male	110 (52.63%)	68 (51.90%)
	Preterm birth		28 (13.40%)	21 (16.03%)
	Term birth		181 (86.60%)	110 (83.97%)
	Weight	Very low	2 (0.95%)	1 (0.76%)
		Low	35 (16.75%)	21 (16.03%)
		Adequate	168 (80.38%)	109 (83.21%)
		Macrosomic	4 (1.91%)	0 (0.00%)
	Respiratory distress syndrome		13 (6.22%)	10 (7.63%)
	Transient tachypnea		33 (15.79%)	6 (4.58%)
	Sepsis		3 (1.44%)	2 (1.53%)
	Necrotizing enterocolitis		1 (0.48%)	2 (1.53%)
	Meningitis		3 (1.44%)	0 (0.00%)

SD, Standard deviation; No, Number of subjects; BMI, Body mass index; GWG, Gestational weight gain; DM1, Diabetes mellitus type 1; IUGR, Intrauterine growth restriction.

When analyzing the distribution of pregestational BMI, based on the classification given by the National Institute of Health (NIH), the pre-pandemic group consisted of 27 (12.92%) with underweight BMI, 144 (68.90%) with normal BMI, 10 (4.78%) with overweight BMI, and 28 (13.40%) obese individuals. In the pandemic group, there were 17 (12.98%) with underweight BMI, 92 (70.23%) with normal BMI, 5 (3.82%) with overweight BMI, and 17 (12.98%) obese individuals (Table 1). Variables were equally distributed (*p* = 0.765, *p* = 0.130, *p* = 0.452, and *p* = 0.055 for underweight, normal, overweight, and obese, respectively). This analysis revealed that pregestational BMI in our adolescent populations was not influenced by the pandemic. This suggests that pregestational BMI is unlikely to act as a confounding variable, indicating that the observed effects are not related to this prior pregnancy variable.

**Figure 1:**
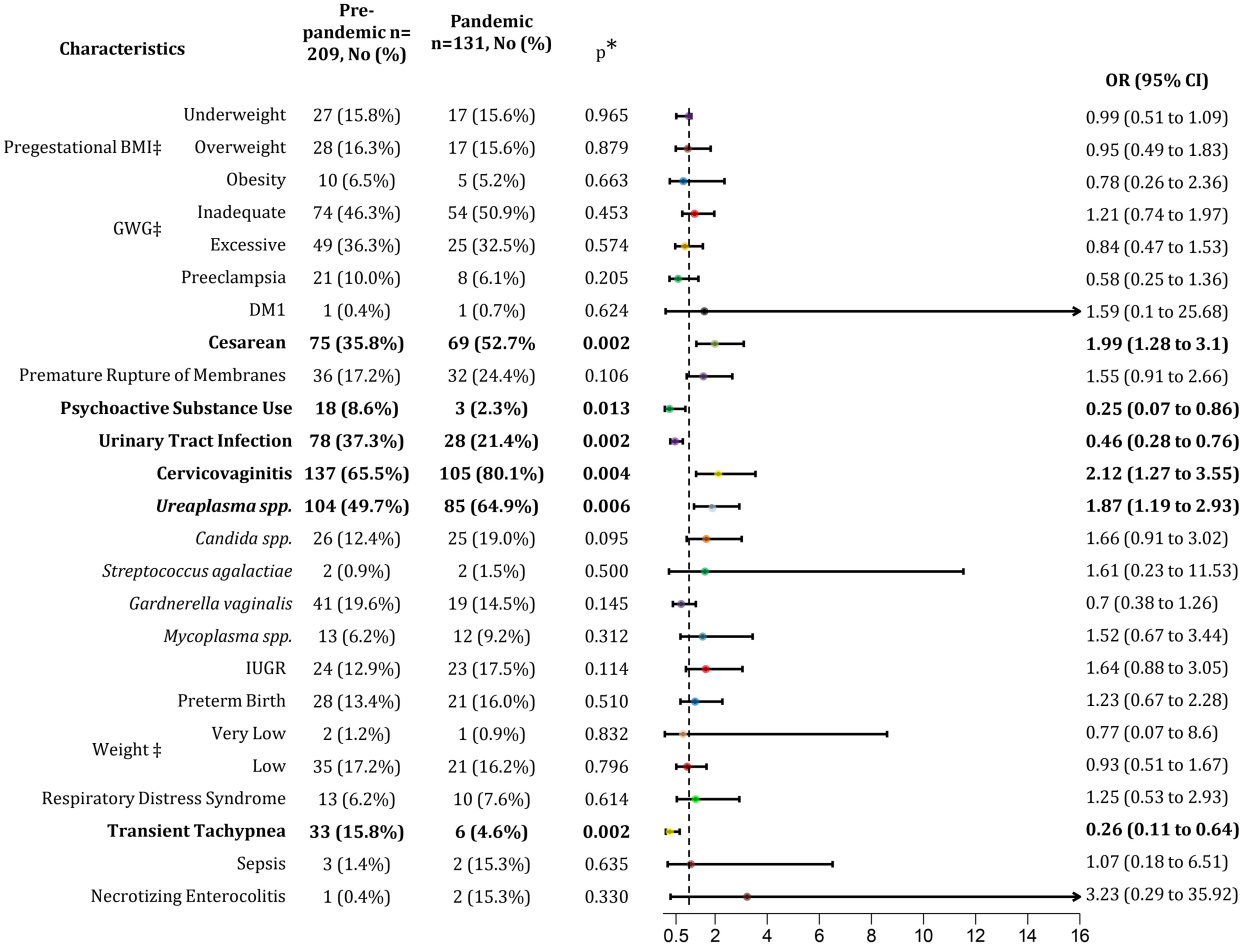
Association of pandemic with maternal and neonatal outcomes. BMI, Body mass index; GWG, Gestational weight gain; DM1, Diabetes mellitus type 1, IUGR, Intrauterine growth restriction; No, Number of subjects; OR, Odds ratio; CI, Confidence interval. *p* < 0.05. ^‡^Percentage calculated respect to normal conditions (normal BMI, Adequate GWG and normal birth weight).

Based on the aforementioned information, we analyzed the distribution of gestational weight gain (GWG) in the adolescent pregnant patients according to the recommendations published by the American College of Obstetricians and Gynecologists (ACOG). The pre-pandemic group consisted of 74 (35.4%) with inadequate GWG, 86 (41.1%) with adequate GWG, and 49 (23.4%) with excessive GWG. In the pandemic group, there were 54 (41.2%) with inadequate GWG, 52 (39.7%) with adequate GWG, and 25 (19.1%) with excessive GWG (Table 1), no statistical difference in gestational weight gain (GWG) between the pandemic and pre-pandemic periods was found (Figure 1). Although we anticipated some differences in gestational weight gain (GWG) between groups, this finding suggest that none of the analyzed variables were related to weight gain beyond the effects of the pandemic.

Maternal outcomes before the pandemic were analyzed, revealing that deliveries were 35.89% cesareans and 64.11% eutocic births. During the pandemic period, the rate of cesarean deliveries increased to 52.67%, while eutocic births decreased to 47.33% (Table 1). This rise in cesarean deliveries was statistically significant, with a notable increase in the odds ratio (*p* = 0.002; OR 1.99; 95% CI 1.28–3.10; Figure 1), indicating that the post-pandemic population had a 66.5% higher risk of undergoing a cesarean section. Additionally, the analysis of gynecological infections acquired during pregnancy revealed a 15% increase in cervicovaginitis cases in the post-pandemic group (Table 1), suggesting that the pandemic influenced the rise in these infections, increasing the risk by 67.9% (*p* = 0.004; OR 2.12; 95% CI 1.27–3.55). Notably, the predominant etiological agent identified was Ureaplasma spp. (*p* = 0.006; OR 1.87; 95% CI 1.19–2.93) (Figure 1). Although the rate of cesarean section deliveries significantly increased during the pandemic, gynecological infections were not associated with this increase.

In contrast to the previous comparison, we observed a 42.73% decrease in urinary tract infection (UTI) cases in the post-pandemic group (from 37.32 to 21.37%; Table 1), with a significant association (*p* = 0.002; Figure 1). This indicates a 68.6% higher risk of developing UTIs in the pre-pandemic group.

During the pre-pandemic period, there was a prevalent population that consumed psychoactive substances during their pregnancies, impacting their overall health. Comparing the pre-pandemic and pandemic groups, we found a 26.59% reduction in psychoactive substance use during the pandemic (8.61 vs. 2.29%, Table 1; Figure 1). Psychoactive substance use was significantly associated with the pre-pandemic period (*p* = 0.013), indicating an 80% higher risk of consumption before the pandemic.

Neonatal outcomes were also analyzed, revealing a slight increase in the preterm births from 13.4% in the pre-pandemic group to 16.03% in the pandemic group, though this difference was not statistically significant. Additionally, the prevalence of transient tachypnea in newborns decreased by 29%, from 15.79% in the pre-pandemic group to 4.58% in the pandemic group (Table 1). As shown in Figure 1, transient tachypnea was significantly associated with the pre-pandemic period (*p* = 0.002; Figure 1), with a 79.4% increased risk.

**Figure 2:**
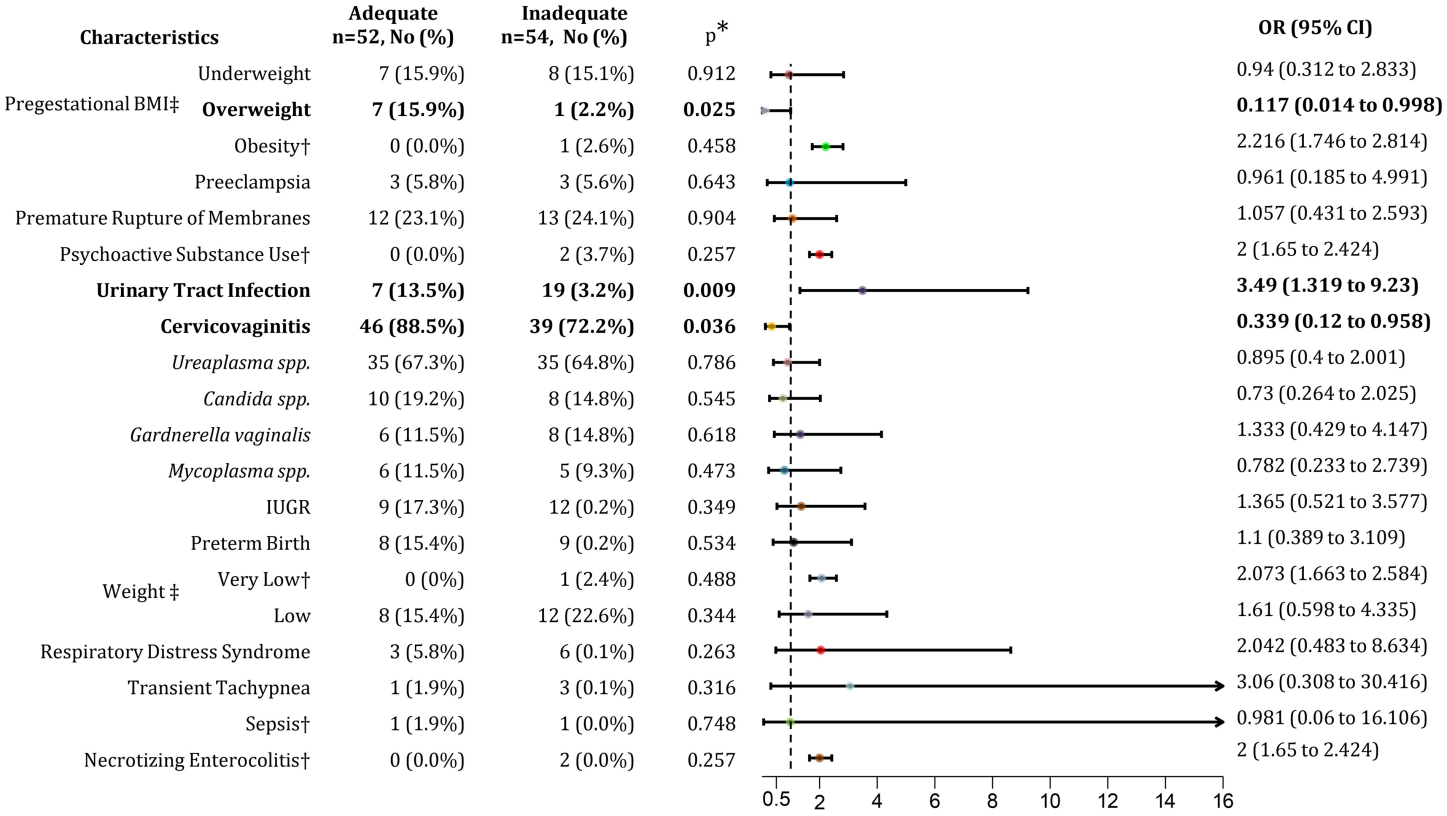
Association of inadequate GWG with maternal and neonatal outcomes. BMI, Body mass index; GWG, Gestational weight gain; IUGR, Intrauterine growth restriction; No, Number; OR, Odds ratio; CI, Confidence interval. ^*^p < 0.05. ^†^Relative Risk (RR) was calculated as OR was not possible to estimate. ^‡^Percentage calculated respect to normal conditions (normal BMI, Adequate GWG, and normal birth weight).

**Figure 3:**
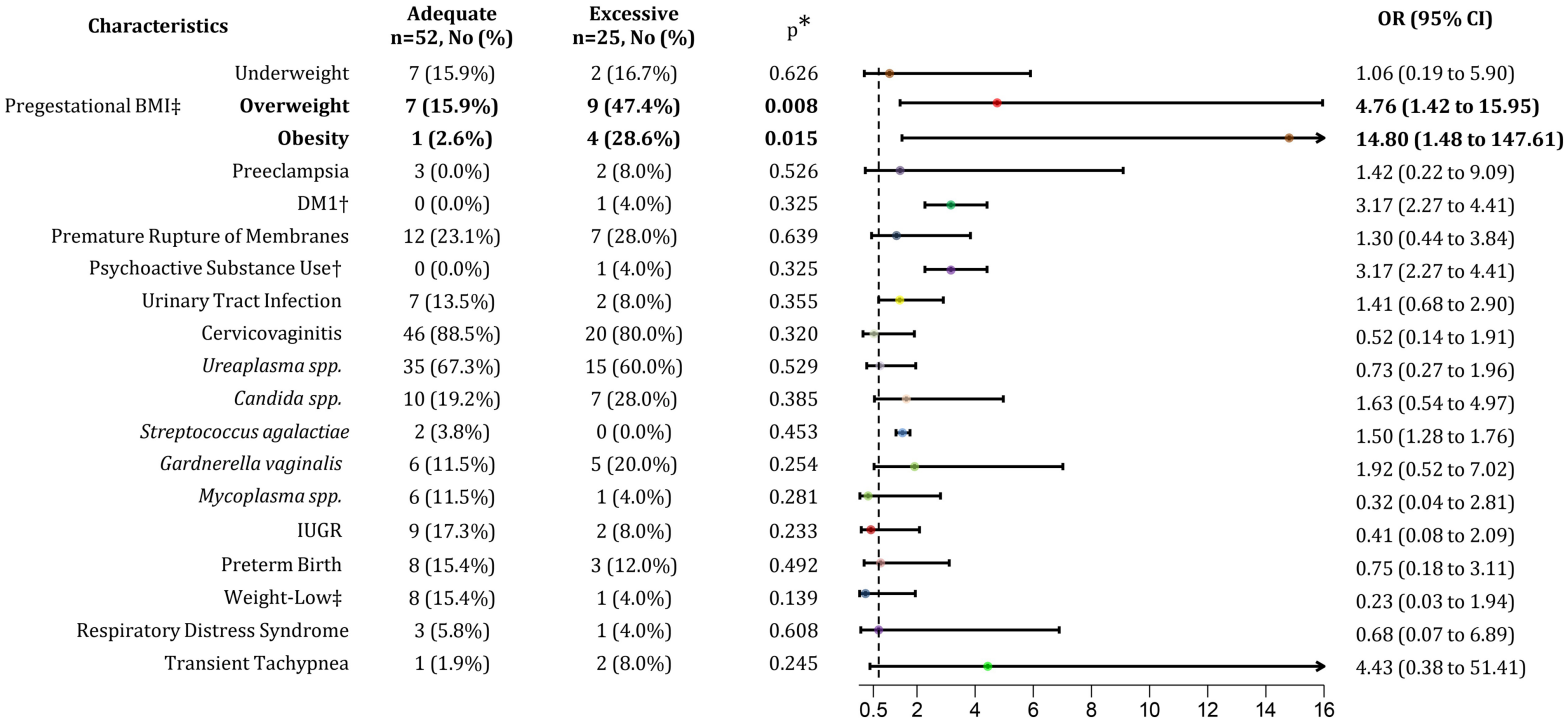
Association of excessive GWG with maternal and neonatal outcomes. BMI, Body mass index; GWG, Gestational weight gain; DM1, Diabetes mellitus type 1; IUGR, Intrauterine growth restriction; No, Number; OR, Odds ratio; CI, Confidence interval. *p* < 0.05. ^†^Relative Risk (RR) was calculated as OR was not possible to estimate. ^‡^Percentage calculated respect to normal conditions (normal BMI, Adequate GWG, and normal birth weight).

In our preceding research, published in March 2024, which focused exclusively on the pre-pandemic group, we analyzed the impact of GWG on the same binomial risk factors and outcomes. We found that being underweight prior to pregnancy significantly increased the risk of inadequate GWG by 80% (*p* = 0.005; OR 4.0; 95% CI 1.45–11.01). It also elevated the risk of certain neonatal outcomes, including intrauterine growth restriction (IUGR) (*p* = 0.017; OR 3.29; 95% CI 1.19–9.09) and low birth weight (LBW) (*p* = 0.002; OR 3.69; 95% CI 1.56–8.73), increasing their risks by 76.6 and 78.6%, respectively (4). Given the significance of these findings, we extended our analysis to the pandemic group, exploring GWG patterns to continue the comparative analysis (Figures 2, 3).

First, we identified factors potentially associated with inadequate GWG. Patients with an overweight pregestational BMI were more likely to achieve adequate GWG, with an 89.4% increased likelihood, suggesting that these patients adhered to nutritional counseling (*p* = 0.025; OR 8.51; 95% CI 1.0–72.35). Additionally, patients with adequate GWG had a 74.6% higher risk of developing cervicovaginitis compared to those with inadequate GWG (*p* = 0.036; OR 2.94; 95% CI 1.04–8.33). Among adolescent pregnant patients with inadequate GWG, we observed a 77.7% increased risk of urinary tract infections (UTI) (*p* = 0.009; OR 3.49; 95% CI 1.32–9.23). No further associations were found between inadequate GWG and other maternal or neonatal risk factors or outcomes (Figure 2).

Comparing patients with adequate and excessive GWG, the distribution of underweight pregestational BMI was similar between both groups, at 15.9 and 16.7%, respectively. However, patients with an overweight pregestational BMI had an 82.6% higher risk of developing excessive GWG (*p* = 0.008; OR 4.76; 95% CI 1.42–15.95). Among those with pregestational obesity, the risk of excessive GWG was even greater, increasing by 93.6% (*p* = 0.015; OR 14.8; 95% CI 1.48–147.61) (Figure 3). These findings suggest that, although overweight prior to pregnancy is associated with achieving adequate GWG, it is also strongly linked to the development of excessive GWG.

Regarding maternal outcomes, including preeclampsia, diabetes (DM1), premature rupture of membranes, and the use of psychoactive substances, no association was found with presenting either adequate or excessive GWG. Similarly, no link was identified with contraction of infections such as UTIs, cervicovaginitis, or those caused by microorganisms like *Gardnerella vaginalis*, *Mycoplasma* spp.*, Ureaplasma* spp., and *Candida* spp. Regarding neonatal outcomes such as IUGR, preterm birth, LBW, respiratory distress syndrome, and transient tachypnea, no correlation was observed with adequate or excessive GWG in the post-pandemic group.

## 4 Discussion

In 2020, the COVID-19 pandemic was declared by the World Health Organization (WHO), changing the paradigm of healthcare delivery as we knew it. In the past few years, there has been a lack of information about the real impact that this pandemic had on the modus operandi of the medical staff and patients during this period, beyond just the infection itself, but how the isolation and preventive measures created a potentially hazardous environment for the increased of prevalence of certain diseases (20).

For a pregnant patient, there should be a constant and thorough healthcare delivery to make sure that the gestation is developing without any problems. To achieve this the mothers of uncomplicated pregnancies are supposed to attend to more than 10 checkups throughout the pregnancy: one each 4 weeks the first 28 weeks of gestation, then one each 2 weeks until 36 weeks of gestation, and finally one each week until delivery. During these appointments, the obstetrician will make sure that the fetus is growing correctly, and that the mother is adapting correctly. For this, an interdisciplinary team should be assembled to conduct all the necessary tests (blood test, ultrasound, etc.) to give the best possible healthcare to the mother and fetus. For adolescent pregnant patients, due to their high-risk status, a bigger team should be assembled, including a maternal-fetal medicine specialist that will make sure that both the fetus and the mother are out of danger (11, 12).

The shift in healthcare delivery paradigm, also changed the way these follow-ups were conducted through in-person appointments. These changes impacted in the recommendation for these prenatal visits, ensuring that there would be just the minimum and necessary contact between the pregnant patients and the healthcare professionals. The American College of Obstetricians and Gynecologists, recommended alternate or reduced prenatal care schedules, decreasing the number of in-person visits to 5 visit for uncomplicated patients, making the possible infection transmission as low as possible.4 Even with these recommendations, the adolescent pregnant patients are considered a high risk group that needs to have more than those appointments to make sure that there is no hazard in that group. Because of this, there were many changes in healthcare delivery and the follow-up due to the lockdown.

Our study included 340 adolescent pregnant patients that receive prenatal care and had their deliveries at the INPer, 209 as part of the pre-pandemic group and 131 as part of the pandemic group. This study focused on creating a comparative analysis of the risk factors and maternal and neonatal outcomes to see if the patterns stayed the same among the different pandemic periods, or if there were changes, to analyze those shifts.

Our study showed a statistically significant increase in the cesarean deliveries in the pandemic group, compared to the pre-pandemic group (*p* = 0.002; OR 1.99; 95% CI 1.28–3.10). Some of the relative and absolute indications for cesarean section include, but not limited to, failure to progress in labor, abnormal fetal heart rate, multiple gestation, placenta previa, placenta abruption, macrosomia, and breech presentation, among others (21). Gurol-Urganci et al. conducted an observational study during the pandemic in England that showed an increase in the rates of cesarean deliveries among pregnant patients, both elective (*p* < 0.001; OR 1.08; 95% CI 1.07–1.09) and emergency procedure (*p* < 0.001; OR 1.10; 95% CI 1.09–1.11) (22). Also in Australia, in a study conducted in west Sydney, results showed a 4.1% increase in cesarean deliveries during the pandemic. When analyzing the kind of procedure, both elective and emergency cesarean increased, by 1.7 and 2.3%, respectively. Overall, there was a higher adjusted relative risk ratio (aRRR), indicating an increased risk of emergency cesarean of 1.36 (*p* < 0.001; aRRR 1.36; 95% CI 1.27–1.45) and elective cesarean of 1.11 (*p* = 0.004; aRRR 1.11; 95% CI 1.04–1.20). When they analyzed the possible reasons behind this increase, they found an increase prevalence in nulliparous, obese women, South Asian ethnicity, and women attended at small hospitals (23). At our institute, we handle high-risk pregnancies, resulting in a higher cesarean rate compared to other maternity hospitals in Mexico. Moreover, during the pandemic, our cesarean rate increased further because of the frequency of maternal complications as preeclampsia and its relation with neonatal outcomes as preterm delivery (PTD), as it was described for adult patients (24). Interestingly, in our adolescent cohort, the prevalence of preeclampsia was lower during the pandemic, yet the rate of cesarean deliveries was higher. This suggests that factors beyond traditional maternal complications may have influenced the decision to perform cesarean sections, potentially due to the challenges and uncertainties posed by the pandemic, as well as the heightened caution in managing pregnancies during this period.

Contrarily, in Switzerland, Cincera et al. found that cesarean delivery during the pandemic period, specifically during the first wave, was 30% lower than during the pre-pandemic period (*p* = 0.004; OR 0.68; 95% CI 0.55–0.84). This change was attributed to the different integration of the obstetrics teams, the change in behavior of the obstetric team and the patients (25). In another study conducted in Nigeria, results showed that there was a decrease in rates of cesarean deliveries during the pandemic, with a decrease of 4.6% compared to the pre-pandemic period during the first wave (*p* = 0.027; OR 0.69; 95% CI 0.63–0.97). Even though in this study statistically significant increase in rate of some adverse outcomes, such as fetal distress, postpartum anemia, emergency cases and post-term deliveries, information is lacking to associated it to the cesarean or vaginal deliveries (26).

Variations between our findings and those of the mentioned studies may be attributed to cultural and socioeconomic factors specific to Mexico. Regarding the violence that women experience in Mexico, The National Institute of Statistics and Geography (Instituto Nacional de Estadística y Geografía; INEGI) conducted the National Survey on the Dynamics of Relationships in Homes (ENDIREH) in 2021. The survey revealed that out of Mexico’s 128 million people, 65.5 million were women (51.2%), and 50.5 million of them (77.1%) were 15 years old or older. Among these women, 70.1% had experienced violence at least once in their lives, whether psychological, economic, patrimonial, physical, sexual, or in the form of discrimination. Psychological violence had the highest prevalence (51.6%), followed closely by sexual abuse (49.7%). Compared to data from 2016, there was a 4% increase in the overall violence women experienced throughout their lifetimes (27).

This alarming rise in violence is particularly concerning as it tends to isolate women, making them less likely to seek help. For adolescent pregnant patients, this creates an additional layer of vulnerability. The combination of pregnancy and youth already place these young women in a fragile position, and the presence of violence can exacerbate their isolation, making it even more challenging for them to access the healthcare and support they need. Addressing this issue is crucial, as the well-being of both the mother and the unborn child is at stake. Comprehensive strategies must be implemented to protect these young women, ensure they receive adequate care, and provide them with safe avenues to seek help (27).

Violence significantly increases in low-income areas, along with substance use. The poorest individuals face challenges in accessing healthcare services, as primary clinics are located in cities, while many of these people live in remote areas. Although our Institute receives high-risk pregnancy patients, we are limited by the number of beds available for mothers and the space for neonates, making it difficult to serve everyone in need of care. Considering the increase in violence during the pandemic and the low-income status of our patients, it is reasonable to assume that they face a higher risk of adverse pregnancy outcomes compared to those in high-income countries or countries that receive substantial attention from international organizations.

Additionally, previous studies have shown that racial and ethnic minority pregnant women, particularly Black and Asian women, as well as those experiencing socioeconomic deprivation, have a higher prevalence of adverse pregnancy outcomes (28). However, Minopoli and collaborators, in a retrospective cohort study conducted in the United Kingdom, found that the burden of adverse pregnancy outcomes was actually higher among white women, who represent the largest group and are significantly impacted by socioeconomic deprivation (29). Another cohort study involving 1,155,981 women demonstrated an increased risk of stillbirth, preterm birth, and small-for-gestational-age births among women from minority ethnic backgrounds who also lived in the most deprived IMD groups (30).

Although these comparisons have not been specifically extended to Hispanic women, it is well recognized that our patients are similarly affected by socioeconomic deprivation. However, there is currently a lack of data specifically addressing these factors in adolescent pregnant patients, underscoring the significance of our study in contributing to the development of this critical information. Regarding educational levels, all of our adolescent patients were enrolled in middle or high school, but we do not know how pregnancy or its outcomes might affect their school attendance and continuity.

Substance use is a prevalent problem around the world, impacting in the health of pregnant and non-pregnant individuals, with a special risk in adolescent patients (31, 32).

In our study, we found a lower incidence of psychoactive substance use during the pandemic, with a higher risk before the pandemic (*p* = 0.013; OR 4.02; 95% CI 1.16–13.93). This pattern was also seen in a study conducted in Canada, which reported substance use during pregnancy of 6.7% for alcohol, 4.3% for cannabis, 4.9% for tobacco, and 0.3% for illicit drugs, which were lower or similar to previous research done in North America before the pandemic. When they analyzed factors for substance use, the results showed that pregnant patients who consumed cannabis and tobacco had symptoms of depression (*p* < 0.008 vs. <0.001), financial difficulties (*p* < 0.001 vs. *p* < 0.001), and threat of baby life (*p* = 0.010 vs. p = 0.005); specifically for cannabis use, not receiving consistent prenatal care (*p* = 0.003). For alcohol with other substance co-use it showed higher use in patients with symptoms of depression (*p* < 0.001), financial difficulties (*p* < 0.001), not receiving constant prenatal care (*p* < 0.001), and constant social isolation (*p*< 0.001) (32). In our study, the depressive and anxious symptoms and disease diagnosis was not correlated as a risk factor for the substance use in adolescent pregnant patients. In previous research conducted at our institute, we observed an increase in depression and anxiety symptoms among adult postpartum patients during the pandemic compared to the period before lockdown, even though we did not explore substance abuse (33). Unfortunately, we do not have observations of this phenomenon in adolescents. It is possible that substance abuse might have decreased due to pregnant adolescents being in isolation with their families or facing difficulties in obtaining drugs. However, it could also be due to incomplete analysis during their pregnancy visits, which were reduced as clinical attention was redirected toward COVID-19 care. Additionally, psychological care providers were focused on managing patients’ stress when SARS-CoV-2 was detected in their babies.

Conversely, the study conducted by Lien et al., which focused on pregnant patients in Memphis, Tennessee, in the United States of America, showed an increase of 11.6% for fentanyl use (*p* < 0.001) and 30.1% for tobacco use (*p* < 0.001) during the pandemic, compared to the pre-pandemic period (34).

Regarding the contraction of infections there was a higher risk in the pre-pandemic group to develop UTI (*p* = 0.002; OR 2.19; 95% CI 1.32–3.62), while there was a higher risk to develop CVV in the pandemic group (*p* = 0.004; OR 2.12; 95% CI 1.27–3.55). Werter et al., in a study on the Netherlands´ pregnant population, showed that during the first lockdown of the pandemic there was no change in the prevalence of UTIs compared to the 2017–2019 period, with a 2.5% decrease during the pandemic (2020) (*p* = 0.61). In the inter-lockdown and second lockdown (2020–2021) there was no statistically significant increase in positive urine cultures for the diagnosis of UTI, with increases of 2.3 and 4.8%, respectively, compared to the pre-pandemic period (*p* = 0.35 and *p* = 0.09) (35). In another study conducted in the Kingdom of Bahrain, results showed more pregnant patients treated for UTI after the start of the COVID period, with a 12.5% increase in cases (*p* < 0.0001). These changes corresponded mainly to changes in practices of public healthcare and not private care delivery (36).

Regarding the cervicovaginits, there is no data yet comparing the total number of cases of CVV between the pre-pandemic, and pandemic/post-pandemic period. In our study, we analyzed the prevalence of different bacterial vaginal infections such as *Ureaplasma* spp.*, Candida* spp.*, Streptococcus agalactiae, Gardnerella vaginalis* and *Mycoplasma* spp. The results showed an increased prevalence of *Ureaplasma* spp. infection with a 15.2% increase in the post-pandemic group (*p* = 0.006; OR 1.89; 95% CI 1.19–2.93). Bahaa et al. also showed a higher prevalence of bacterial vaginosis infections treated during the pandemic in the pregnant population of Bahrain, with a 9.7% increase (*p* < 0.0001) (36).

In another study conducted by Hao et al. on pregnant patients in Beijing, there was an increase in cases of cervicovaginitis in the pandemic group, with a 4.7% increase (*p* < 0.001). The main agents identified were candidiasis vaginitis and mycoplasma vaginitis. Candidiasis vaginitis had a higher prevalence during the pandemic, with a 4.48% increase compared to the cases before the pandemic, although these results were merely observational and not statically significant (*p* = 0.055). When the confounding factors were taken into consideration, there was no significant change in the cases of vaginitis (without controlling confounders—*p* < 0.001; OR 3.15; 95% CI 1.86–5.35 vs. Controlling confounders *p* < 0.001; OR 3.29; 95% CI 1.88–5.78). This study did not find a direct cause for this increased incidence of cases (37). Regarding *Ureaplasma* spp., there are no studies yet published in the literature that compare its prevalence as a cause of vaginal infections before, during and after the pandemic in pregnant patients. However, Akinosoglou et al. showed increasing rates of antibiotic resistance for *Ureaplasma* spp. from 2014 to 2022, specifically to clindamycin and erythromycin, in a Greek population, suggesting a possible cause for more cases with increase antibiotic resistance (38). Analyzing the patterns seen in our population, higher rates of UTI and CVV, may had been caused by different factors such as different urogenital microbiome making them more prone to contraction of these infections (5), fear of seeking medical assistance during the pandemic, delaying treatment until the cases were advance and require medical assistance, and decrease hygiene due to psychological distress and socioeconomic problems. The rise in UTIs prior to pandemic may be linked to substances abuse, which was also observed at higher rates before COVID-19 pandemic. Substance abuse has been demonstrated to dysregulate immune response, making patients more susceptible to infections (39, 40). Conversely, CVV was more frequent during and after pandemics. In this regard, Celik et al. demonstrated dysbiosis in the vaginal microbiota of COVID-positive pregnant patients, possibly contributing to the increase in CVV infection during the pandemic (41).

In the case of neonatal outcomes, our study showed a higher prevalence of newborn transient tachypnea before the start of the pandemic (*p* = 0.002; OR 3.87; 95% CI 1.57–9.52). Ferrara et al. studied the pregnant and newborn population from Oakland, comparing the outcomes before and during the pandemic. Their study showed no change in prevalence and risks of transient tachypnea before and during the pandemic. The authors divided their groups into three based on the time of exposure to the pandemic: T1 were those unexposed (delivery from July 1, 2018, to February 29, 2020), T2 were those partially exposed (delivery from March 1, 2020 to December 5, 2020) and, T3 were those fully exposed (delivery from December 6, 2020 to October 31, 2021). When they analyzed the implantation for a multimodal prenatal health care model (in-office and telemedicine healthcare delivery), they showed statistical significance when the pandemic period was compared with the pre-pandemic period. The T2 group had lower cases of tachypnea compared to the T1 group (*p* = 0.03) and the T3 group have less tachypnea cases than the T1 group (*p* = 0.002), even though there was no increased risk for the development of this pathology in the pre-pandemic group (42).

When analyzing the possible reasons for this, we reviewed some studies that compare different factors as possible risk factors. A study conducted in Poland reviewed clinical characteristics of newborns from COVID-19 infected mothers. The results showed that neonates from these mothers were at higher risk that non-infected mothers to have tachypnea (*p* = 0.037; OR 3.27; 95% CI 1.02–10.5). Upon analyzing the data, it was found that even though the most common respiratory insufficiency in the infected group has tachypnea, the reason for need of non-invasive ventilation and development of these cases was not associated to the infection itself, but with factors such as prematurity, transient tachypnea, and respiratory distress (43). In another study conducted by Vardhelli et al., it was shown that even when one of the most frequent symptoms SARS-CoV-2 in newborn was the respiratory distress, the need for non-invasive ventilatory support was due to other causes such as prematurity, transient tachypnea, and respiratory distress syndrome, rather than from the COVID pneumonia (44).In our study we did not include maternal COVID-19 infection as a criterion. This decision stemmed from lack of available diagnosis techniques at the beginning of the pandemic, changing protocols regarding who to test, and subsequent subclinical cases that were not tested later in the pandemic when testing was no longer obligatory for every mother entering the maternity ward. A plausible explanation for the pattern seen in our population is that neonatal resuscitation techniques and the care provided to newborns immediately after delivery were more rigorous and thorough during the pandemic. This increased level of care may have facilitated better extra-uterine adaptation, resulting in fewer cases of newborn transient tachypnea. Also, COVID vaccination have been studied to determine their impact on neonatal outcomes. In India, a study conducted at a tertiary health center examined the outcomes of vaccination in pregnant women. They found no significant differences in the risk of adverse outcomes between the vaccinated and unvaccinated groups (45). In a systematic review and meta-analysis by Hameed et al., it was found that COVID-19 vaccination was safe and did not increase or shift the prevalence of adverse perinatal outcomes, such as low APGAR scores (indicating problems during extra-uterine adaptation), miscarriages, stillbirths, babies born small for gestational age (SGA), cesarean sections, or prematurity (46).

Derived from the results found in our previous study, analyzing the impact of pre-gestational BMI and GWG in mother and neonate outcomes before the pandemic, we decided to analyze the pattern focusing on the pandemic group (8). In our study, the pandemic adolescent pregnant patient with overweight pre-gestational BMI showed a high prevalence and increased rates of adequate and excessive GWG when comparing adequate with inadequate GWG and adequate with excessive GWG, respectively (*p* = 0.025; OR 8.51; 95% CI 1.0–72.35 vs. *p* = 0.008; OR 4.76; 95% CI 1.42–15.95). Meanwhile, the patients that showed an obesity category of pre-gestational BMI had a higher risk of developing excessive GWG (*p* = 0.015; OR 14.8; 95% CI 1.48–147.61). Contrary to the findings that we found in our previous studies, which showed a high prevalence of underweight pre-gestational BMI corelated with inadequate GWG, the pandemic group did not have an increased risk for developing inadequate GWG in any of the of pre-gestational BMI categories (8).

These results showed a pattern discrepant with other studies such as the one conducted by McPhail et al. in TRICARE beneficiaries. The overall results showed increased rates of excessive GWG pattern in the pre-pandemic group, although it did not show statistical significance; in most results, there were no significant differences in GWG when comparing pre-pandemic and pandemic periods. They attributed this to the kind of population studied, even though previous studies have shown increased GWG due to low physical activity levels, unbalanced diet, and high stress in this population (47). Regarding emotional impact on GWG, a study conducted by Zhang et al. in pregnant population in various regions of China found that patients with higher emotional eating (EE) scores due to stress had higher risk of excessive GWG (OR 1.9; 95% CI 1.08–3.32). The EE in these patients was associated with higher consumption of foods such as cereals and oils and decreased in fish and seafood (48).

In our adolescent pregnant, the patients received psychological and nutritional guidance. We infer that those with pre-gestational BMI categorized as overweight had a better adherence to the integrative health plan created for them, resulting in adequate GWG compared to the patients in the pre-pandemic group (8). With more time in their hand during the pandemic, more meals at home, and less peer pressure and potential unhealthy dietary options, they were able to gain the weight needed for a healthy pregnancy. Even though thinking that every patient strictly followed the psychological and dietary is tempting, we acknowledge the reality of our population and the eating habits in Mexico, which include a high processed food and simple carbohydrate diet. Combined with the stress of the pandemic, this resulted in patients with previous deleterious eating habits marked by their overweight and obesity pre-gestational pattern, continue to have poor eating habits and EE during the pandemic, leading to excessive weight gain.

Finally, concentrating on the infection contraction, in our previous study there was no increased risk of infections, such as UTI and CVV, related to the GWG (8). However, in this study, we found an increased risk of UTI in adolescent pregnant patients with inadequate GWG compared to those with adequate GWG (*p* = 0.009; OR 3.49; 95% CI 1.32–9.23). Rejali et al. studied the GWG patterns along with the UTI contraction and found no correlation between them in the Shahrekord population (49). In the case of CVV there was a higher risk in those with adequate GWG in comparison to those with inadequate GWG (*p* = 0.036; OR 2.94; 95% CI 1.04–8.33).

As it was aforementioned, during the reproductive stage and specially in the adolescence and pregnancy, the changes in the urogenital microbiome make patients more susceptible to develop dysbiosis, and consequently, an increased risk to contract urogenital infections (5). Weight is a known modulator of the immune response, making the patients more prone to develop UTI (50–52). Along with the psychological health caused by the pandemic, impacting on the immune system of our pregnant patient, this may have contributed to the contraction of these infections in our adolescent pregnant patients.

The COVID-19 pandemic significantly altered the global healthcare delivery paradigm, particularly affecting adolescent pregnant populations. This study highlights the profound impacts and shifts in patterns of life and healthcare before, during, and after the pandemic. These findings underscore the necessity for adaptability within healthcare systems to ensure continuous and comprehensive care for pregnant patients, minimizing disruptions to maternal and neonatal health.

Finally, despite the preference of most patients and doctors for face-to-face medical appointments over video telemedicine, it is crucial to develop high-quality alternatives to ensure greater access and better monitoring within the healthcare system. Ultimately, the goal should be the personalization of medicine, whether delivered in person or through telemedicine (53, 54).

### 4.1 Strengths and limitations

The relevance of this study lies in its identification of the significant changes in adolescent pregnant outcomes, due to probable changes in life patterns and healthcare delivery before, during, and after the pandemic. The findings emphasize the critical need for healthcare systems to be adaptable and resilient in the face of such disruptions. By highlighting how the pandemic affected maternal and neonatal health, the study underscores the importance of maintaining consistent and comprehensive care for pregnant patients, even in challenging times; especially in vulnerable populations such as adolescents, who are at risk for adverse outcomes due to inherent metabolic, physiological, psychological, and immunological changes. This research has practical implications for improving healthcare policies and strategies to better protect vulnerable populations in future crises.

One major limitation is selection bias, as the study was conducted at a tertiary care institution that primarily handles high-risk pregnancies. This could mean that the population studied may not fully represent the broader adolescent pregnant population, particularly those with less access to specialized care, potentially leading to an overestimation of complications like cesarean rates and adverse neonatal outcomes. Additionally, several confounding variables, such as changes in healthcare policies, the introduction of telemedicine, and shifts in patient behavior during the pandemic, may have influenced the outcomes independently of the pandemic; we should also consider pandemic-related factors, such as initial SARS-CoV-2 infection, reinfection, vaccination status, the type of vaccine administered, and whether corticosteroid treatment was used. Socioeconomic factors, including access to healthcare and nutritional status, could also differ between the pre-pandemic and pandemic groups, potentially masking the true effects of the pandemic on maternal and neonatal health.

## Data Availability

The original contributions presented in the study are included in the article/supplementary material, further inquiries can be directed to the corresponding author.
